# The financing of medicines in Brazilian municipalities: whose responsibility is it?

**DOI:** 10.11606/s1518-8787.2024057005565

**Published:** 2024-11-25

**Authors:** Wendell Rodrigues Oliveira da Silva, Rodrigo Fonseca Lima, Ivanessa Thaiane do Nascimento Cavalcanti, Rafael Santos Santana, Silvana Nair Leite

**Affiliations:** I Universidade de Brasília. Programa de Pós-graduação em Ciências Farmacêuticas. Brasília, DF, Brasil; II Universidade Federal de Santa Catarina. Programa de Pós-graduação em Assistência Farmacêutica. Florianópolis, SC, Brasil

**Keywords:** Pharmaceutical Assistance, Capital Financing, Primary Health Care, Essential Medicines, Cities

## Abstract

**OBJECTIVE:**

To analyze the investments made in medicines by the federated entities and the asymmetries in these investments from 2016 to 2020, which may have an impact on the supply of and access to these medicines in the SUS.

**METHODS:**

This is an exploratory, retrospective study to identify who are the main entities responsible for investment in Primary Care medicines in municipalities, the evolution, counterparts, and regional differences of this investment between 2016 and 2020.

**RESULTS:**

The amounts spent on medicines by Brazilian municipalities were higher than the contribution to the CBAF from the MS or the MS + State in all the years analyzed. The average percentages of federal funds transferred and municipal spending varied according to the region of Brazil. The average *per capita* amount invested in medicines by municipalities increased between 2016 and 2020 (deflation applied), with a greater impact for municipalities with lower MHDI. The Farmácia Popular program mainly reaches municipalities with the largest populations and the highest MHDI and is therefore not enough to address the inequalities in access pointed out.

**CONCLUSIONS:**

There has been a widening of inequalities in the capacity of municipalities to ensure access to medicines, especially among the most vulnerable municipalities, accumulating even more risks of illnesses and deaths from primary care-sensitive diseases.

## INTRODUCTION

Ensuring access to medicines considered essential is fundamental for resolving health issues in primary care. According to the World Health Organization (WHO), the lack of access to medicines causes a cascade of misery and suffering and even deaths from diseases that are preventable or curable^1^.

Financing the purchase of medicines is a topic of great interest in all countries and different health systems^2^. The Unified Health System (SUS – *Sistema Único de Saúde*) is responsible for access to medicines. The Basic Component of Pharmaceutical Services (CBAF – *Componente Básico da Assistência Farmacêutica*) is intended for the acquisition of medicines and supplies set out in Annex I and Annex IV of the National List of Medicines (Rename – *Relação Nacional de Medicamentos*), including those related to health problems and primary care programs.

The resources for funding the CBAF are shared on a tripartite basis, and the management of these resources is the responsibility of the municipalities. The *per capita* amounts transferred from the Ministry of Health (MS) and the states to the municipal health fund are defined in MS ordinances. In 2017, the amount set for transfer from the MS was R$ 5.58 per inhabitant/year^3^. In 2019, Ordinance No. 3,992/2017 was published, which changed the way resources are funded and transferred to health actions and services in two blocks: the Health Actions and Services Funding Block and the Health Network Investment Block. CBAF funding continued to be defined by specific ordinance. This year, the Municipal Human Development Index (MHDI) was used to define the amounts to be passed on by the MS to the municipalities to fund the CBAF, which vary from R$ 5.85 to R$ 6.05 and are still in force until 2023 with no readjustment^4^. The MS is therefore responsible for around 50% of the minimum amount stipulated for the cost of the medicines made available by the municipalities. The states and municipalities are responsible for smaller *per capita* portions of the total CBAF budget.

In Brazil, it is possible to see asymmetries in *per capita* investments in PS: from 2010 to 2019, there was an increase in this value in the Midwest, Southeast, and South regions, while the Northeast and North regions invested less than the national average and showed a decrease in the value of *per capita* investment in medicines^5^. The lower application of financial resources for PS in some regions has already been identified and is a cause for concern, which has not yet led to specific policies capable of mitigating inequities^6^. Between 2005 and 2009, there was an overall increase of 61.6% in SUS resources for the purchase of medicines; in this period, the states and the Federal District increased the volume of their own resources allocated to financing the purchase of medicines by 112.4%, while the municipalities recorded an increase of 22.7% in the Public Health Budget (SIOPS)^7^. The amount invested in 2009 was still considered lower than the *per capita* amount invested in medicines in countries like Canada and Italy^8^.

Pontes et al.^6^, analyzing the data recorded by municipalities in the National Database of Pharmaceutical Services and Actions, found that those in the Southeast, on average, applied a higher amount per inhabitant/year and purchased more items than those in other regions. However, only 17% of Brazilian municipalities sent qualifying data.

It is also important to consider that, in a survey of a national sample of 600 municipalities^9^, 35.4% of municipal health secretaries stated that they use CBAF medicine resources to cover the demand for other medicines, and only 9.7% stated that these resources are sufficient to meet the demand for primary care.

The structuring of PS in Brazilian municipalities, in addition to direct investments in the medicine acquisition, also presents unequal conditions between geographic regions, including physical structure, workforce, management capacity^9^, resulting in inequality in the medicine supply made available to the population between these regions^14^. Even so, 59.8% of users of primary care units reported, in 2015, that they had full access to medicines prescribed by the SUS, demonstrating the great social impact of PS in primary care^15^.

Financing the medicine purchase and ensuring accessibility in primary care are even more critical issues today, as data from the 2019 National Health Survey revealed that only 30.5% of people obtained their medicines from the SUS, on average nationwide, with the South Region having the greatest access^16^. This result is especially worrying as it reveals that people’s access to medicines may have been decreasing in recent years, considering that, in a 2014 national survey, 47% of the Brazilian population stated that they obtained their prescribed medicines for chronic diseases from SUS units^17^. In recent years, an 18.24% readjustment in medicine prices has also been authorized by the Drug Market Regulation Chamber (CMED, 2016 to 2020) in parallel with the economic recession that has hit the population and led to greater dependence on the SUS.

Throughout the history of the consolidation of the SUS, PS has become a fundamental policy and service with a major impact on the Brazilian population^18^. In addition to the regulation of the financing of medicines by component, the Farmácia Popular Program was set up in the SUS to expand the capacity to promote access to medicines for prevalent conditions, which has consolidated itself as an important strategy for guaranteeing rights for the population. Nowadays, when the primary health care model and its financing are undergoing constant and worrying changes^19^, and when public policies for equity and universal access to health in the country are being resumed, it is necessary to analyze how the acquisition of medicines for the health conditions treated in primary health care services has actually been financed, seeking to identify possible inequities, distortions and inadequate financing conditions for the population, access to medicines, and threats to the sustainability of municipalities as primary health care managers.

The transfer of resources to municipal health management is a critical factor to be analyzed. Issues such as high income inequality and social development, large territorial extensions and regions with difficult access, and regional contrasts increase the degree of difficulty in providing basic care services, including PA20. The aim of this study was to analyze the resources used to purchase medicines for primary health care among the responsibilities of the federated entities over a recent five-year period (2016, 2018, and 2020), which includes the time when a new calculation for the transfer of federal resources was applied. The study covers all Brazilian municipalities based on SIOPS records, analyzing the characteristics of municipalities as managers of these resources and the impact of the counterparts agreed in the tripartite.

## METHODS

This is a retrospective exploratory study, which identifies and compares the evolution of the values of the CBAF counterparts by the MS, states, and municipalities, in the time series of 2016, 2018, and 2020. The period includes transfers before and after the corrections to the value of the counterpart by the MS in 2017^3^.

The data on the amounts of the MS’s contribution was collected from the National Health Fund (FNS) website^21^ through the statement of transfers of resources made by this body, by funding block. Of the 5,568 municipalities surveyed, it was found that only the municipality of Cárcere/MT did not receive transfers of funds from the MS in the years surveyed and Brasília was not included in the survey because it is not a municipality. All the funds decentralized by the FNS for the purchase of CBAF medicines (directly to the municipalities or to the states for centralized purchase) were counted for each municipality of destination. We tried to identify the regularity of the transfer of CBAF funds from the states to the municipalities in various ways (by contacting sectors of the MS, the National Council of Health Secretaries (Conass), and the National Council of Municipal Health Secretaries (Conasems)), but we were unable to find a reliable source of information on this transfer for all Brazilian states. In this way, the study considered that all the funds relating to the states’ counterpart, as provided for in the regulations in force each year, had been passed on.

The data on the population, total spending on health, and the percentage of spending on medicines was taken from the SIOPS Information System^22^, which is a tool for monitoring compliance with the constitutional provision that determines, in the budget, the minimum application of resources to public health actions and services:

- Population, which represents the population of the municipality as published by the Brazilian Institute of Geography and Statistics (IBGE), based on the census and its annual estimates;
- Total health expenditure (Total Health E.), which represents the total expenditure on health by the entities during the period evaluated. This indicator is the result of spending on health, per inhabitant, from all sources, whether taxes, transfers from the SUS (Union, states, and other municipalities), credit operations, and others;
- Percentage share of expenditure on medicines in total health expenditure, whichrepresents the share of expenditure on medicines in total health expenditure (% Medicines E.).

Spreadsheets were drawn up covering all Brazilian municipalities, allowing analysis based on absolute and relative frequencies over the time interval examined, using secondary data from the FNS and SIOPS. The statistical program *Rstudio* was also used to clean outliers from the database, according to the box plot and the percentage (1% and 99%).

To calculate the amount invested in medicines by the municipalities, data on total health expenditure was used and multiplied by the percentage share of medicines expenditure in total health expenditure.

The *per capita* amounts spent on medicines per region were calculated, taking into account the average of the years of analysis; the average of the percentage of the transfer of resources from the MS and the MS resource plus the state counterpart in the expenditure on medicines; the average of the value of the three years of the transfer of resources from the MS and the MS resource plus the state counterpart in the expenditure on medicines and the average of the amount spent on medicines in 2016, 2018, and 2020.

The amounts spent on medicines and federal and state transfers to municipalities were monetarily updated. These amounts were deflated for December/2021, using the annual variation of the Extended National Consumer Price Index (IPCA), calculated by the IBGE and obtained from its website adjusted by the population estimate for each year analyzed. The option to use this deflator was based on Law 10,742/2003, which established the rules for regulating the pharmaceutical sector and defined this index for the purposes of adjusting the prices of medicines in the country.

Due to the self-declaratory nature of the SIOPS, it was not possible to identify, in relation to the municipalities’ expenditure, which amounts were paid with funds from the Union, the state, or the municipality itself.

The data on pharmacies registered with the Farmácia Popular Program was extracted from the Farmácia Popular - Management 2.0 system, which is responsible for integrating the pharmacies accredited to the MS.

Regarding population size, the stratum model developed by the Qualifar-SUS Program, an MS program that supports PS activities in Brazilian municipalities, was used for those with up to 25,000 inhabitants, from 25,001 to 50,000, from 50,001 to 100,000, from 100,001 to 500,000, and municipalities with more than 500,000 inhabitants^23^.

The MHDI index estimates the human development of a municipality and classifies municipalities into five bands: very low (from 0.000 to 0.499), low (0.500 to 0.599), medium (0.600 to 0.699), high (0.700 to 0.799), and very high (from 0.800 onwards)^24^.

The *Student’*s t-test was used, with results of p < 0.05 (5%) being considered significant. In this sample, the T-test for two independent (or unpaired) samples was used to compare the means of two independent samples.

## RESULTS

**Figure 1:**
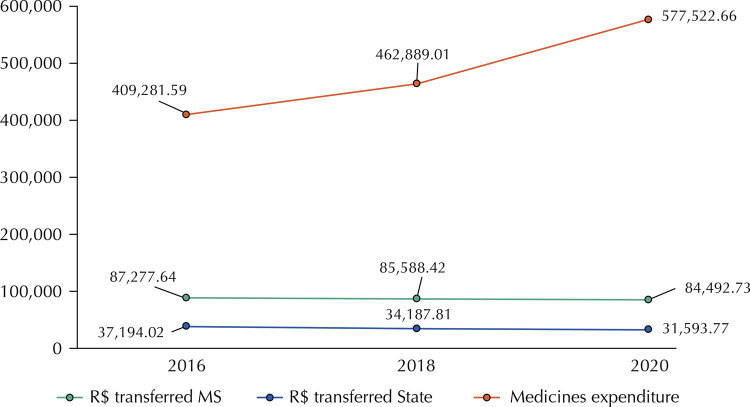
Median spending (R$) on medicines by Brazilian municipalities and on transfers made by the Union (MS) and States in 2016, 2018, and 2020.

Regarding funding for the purchase of medicines, the amounts spent on medicines by Brazilian municipalities were higher than the amounts of the MS’ counterpart or the MS + State’s counterpart (for CBAF funding) in all the years analyzed. The median spending on medicines by municipalities (n = 3,740) went from R$ 409,281.59 in 2016 to R$ 577,522.66 in 2020 (n = 4,152), representing a 41.1% increase in median spending. In relation to the median financial transfer from the MS and the state to the municipalities, there was a reduction of 3.19% and 15.01%, respectively, when comparing the years 2016 and 2020, and an inflationary adjustment was applied (Figure 1).

**Figure 2:**
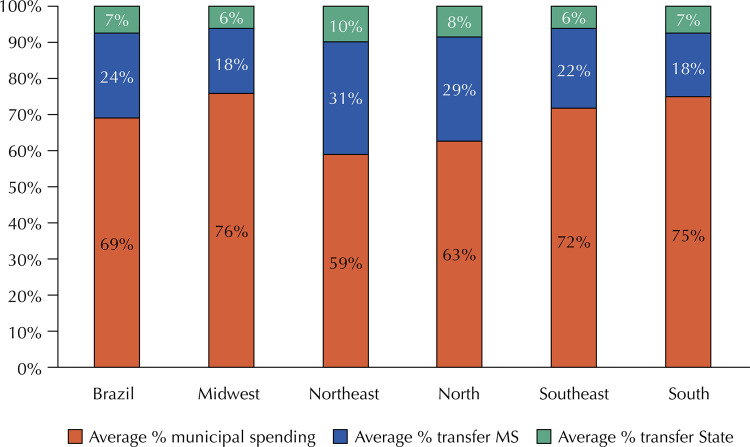
Average percentage (%) of transfers and municipal spending by region.

The average percentages of federal funds transferred with and without state counterparts and of municipal spending over the three years analyzed vary according to the region of Brazil (Figure 2). The national average percentage of municipal spending was 69, ranging from 59 in the Northeast to 76 in the Midwest.

Table 1 shows that the average percentage of funds transferred by the MS and the states over the average total amount invested in medicines by Brazilian municipalities in 2016 and 2020, by population stratum and MHDI, decreased significantly (p < 0.05) in the vast majority of municipalities. Only in municipalities with 50,001 to 100,000 inhabitants and very high MHDI was there no drop in the representativeness of the MS transfer, as shown in Table 1.

**Table 1:** Analysis of the average percentage of resources transferred by the MS over the total amount invested in medicines by Brazilian municipalities in 2016 and 2020, by population stratum and MHDI.

Population stratum (inhabitants)	MHDI	n	2016		2020		Difference from 2016 to 2020	
			n	% of total	n	% of total	%	p-value
Up to 25,000	Very low	25	16	42	16	29	13	0.024
	Low	1,111	599	48	655	27	21	0.0398
	Medium	1,778	1,166	33	1,336	22	11	0.016
	High	1,209	969	23	1,064	18	5	0.0041
	Very high	2	2	23	2	7	16	-
25,001 to 50,000	Very low	9	6	51	7	19	32	0.0142
	Low	204	125	65	135	35	30	0.0248
	Medium	269	154	49	179	33	16	0.0285
	High	279	228	34	247	28	6	0.0035
	Very high	3	2	17	2	12	5	0.0198
50,001 to 100,000	Very low	2	0^a^	-	1	74	-	-
	Low	47	30	60	33	39	21	0.0198
	Medium	124	65	60	78	38	22	0.0381
	High	176	136	37	149	30	7	0.0058
	Very high	5	5	22	4	25	-3	0.0051
100,001 to 500,000	Very low^a^	0	0	-	0	-	-	-
	Low	5	3	58	2	37	21	0.0634
	Medium	61	38	54	44	34	20	0.0202
	High	191	144	37	150	35	2	0.0082
	Very high	20	16	25	18	20	5	0.0041
Above 500,000	Very low^a^	0	0	-	0	-	-	-
	Low^a^	0	0	-	0	-	-	-
	Medium	1	0^a^	-	1	37	-	-
	High	34	24	50	19	43	7	0.0283
	Very high	13	12	44	10	34	10	0.0057

MHDI: Municipal Human Development Index; n: number of municipalities.

^a^ No municipality for the specific MHDI classification and/or year.

As can be seen in Table 2, the percentage of municipalities that have at least one pharmacy accredited to the Farmácia Popular Program increases with size and HDI between the strata presented. In addition, there is a statistically significant increase in the average *per capita* amount invested in medicines by municipalities between 2016 and 2020 (deflation applied) in all strata, except for municipalities with more than 500,000 inhabitants with a high MHDI. Apart from municipalities with up to 25,000 inhabitants, those with the lowest MHDI had the biggest increases in the amounts invested over the same period (Table 2).

**Table 2:** Percentage of municipalities with pharmacies accredited to the Farmácia Popular Program and average *per capita* amount invested in medicines in 2016 and 2020 by Brazilian municipalities by population stratum and MHDI.

Municipal data			Municipalities with FP	Data related to per capita value					
Population stratum (inhabitants)	MHDI	n	2020	2016		2020		Difference from 2016 to 2020	
			%	n	Average (R$)	n	Average (R$)	%	p-value
Up to 25,000	Very low	25	20	16	43.95	16	63.06	43	0.024
	Low	1,111	49	599	42.06	655	65.01	55	0.0398
	Medium	1,778	78	1,166	53	1,336	75.68	43	0.016
	High	1,209	92	969	77	1,064	94.38	23	0.0041
	Very high	2	100	2	55.29	2	146.83	166	-
25.001 a 50.000	Very low	9	22	6	28.93	7	60.13	108	0.0142
	Low	204	70	125	26.27	135	50.01	90	0.0248
	Medium	269	93	154	31.36	179	43.14	38	0.0285
	High	279	100	228	44.86	247	56.64	26	0.0035
	Very high	3	100	2	57.2	2	72.93	28	0.0198
50.001 a 100.000	Very low	2	0	0	-	1	12.29	-	-
	Low	47	81	30	23.78	33	40.98	72	0.0198
	Medium	124	97	65	27.53	78	36.55	33	0.0381
	High	176	100	136	39.28	149	49.04	25	0.0058
	Very high	5	100	5	55.6	4	64.05	15	0.0051
100.001 a 500.000	Very low^a^	0	0	0	-	0	-	-	-
	Low	5	100	3	16.88	2	26.57	57	0.0634
	Medium	61	100	38	33.21	44	52.39	58	0.0202
	High	191	100	144	35.68	150	40.17	13	0.0082
	Very high	20	100	16	44.27	18	52.23	18	0.0041
Above 500,000	Very low^a^	0	0	0	-	0	-	-	-
	Low^a^	0	0	0	-	0	-	-	-
	Medium	1	100	0	-	1	24.59	-	-
	High	34	100	24	35.1	19	30.32	-14	0.0283
	Very high	13	100	12	36.05	10	44.12	22	0.0057

FP: pharmacies accredited to the Farmácia Popular Program; IDHM: Municipal Human Development Index; n: number of municipalities.

^a^ No municipality for the specific MHDI classification and/or year.

## DISCUSSION

The median value of the transfer of the MS’ contribution from 2016 to 2020 fell by 3.19%, showing that the MS’ investments in medicines are not being monitored in municipal spending. In the same period, the average *per capita* amount invested in medicines by municipalities increased significantly (deflation applied) in all population strata and especially among those with the lowest MHDI. As a result, the financing of this component has been expanded with municipalities’ own resources, which accounted for 67%, on average, of all the resources invested in the purchase of medicines at the municipal level between 2016 and 2020. Considering, however, the general increase in investment in medicines by the MS over the same period, it is also important to consider that the MS directly invests resources in the purchase of medicines and supplies for primary care, such as insulins and medicines related to the Women’s Health Program. The Programa Farmácia Popular do Brasil, although it does not count towards the amounts used in the Pharmaceutical Services Components, in practice also helps to increase access to standardized medicines for primary care in the municipalities it covers. Even so, the significant increase in direct investments made by the municipalities indicates an increase in the responsibility of these federal entities for guaranteeing the right to access medicines.

The behavior of primary care financing has been largely pressured by the increase in the municipalities’ burden on this account. Araújo^25^ has already shown that, in a scenario that combines decentralization and underfunding of public health policy, Brazilian municipalities are the ones that contribute the most to the health sector in terms proportional to their revenue and significantly increase their spending in the sector, allocating a proportion of their own revenue at levels higher than those determined by the constitution. A recent description shows that overall investment in health by the Union increased by 30.7% in 2019 when compared to 2012; the states showed a slight drop in investment during the same period, while the municipalities showed a greater increase (32.8% from 2010 to 2019)^5^. In the study by De Seta et al.^26^, it was identified that, from 1991 to 2017, the Union’s share of SUS funding fell from 73% to 43%, and was accompanied by an increase in the allocation of resources, mainly municipal, leading to a greater budgetary burden on municipalities.

The current study shows that the behavior of the financing of the purchase of primary care medicines in this period between the federated entities showed a greater discrepancy than that observed in general health financing. The municipalities’ contribution showed an even greater percentage increase in relation to the federal and state counterparts than has been observed in health financing, despite the readjustments to the CBAF transfer amounts regulated in 2017 and 2019.

Bruns et al.^27^ present data, however, which may be an aggravating factor to the results shown here, as they indicate that some states have failed to pass on their state counterparts, showing the low contribution of this sphere to the municipalities. Therefore, considering that this study adopted the assumption that the states had made their standardized contributions (in terms of amounts or medicines), the data presented may be even more alarming for the municipalities. Like a vicious circle, the lack of these resources can influence the increase in demand for medium and highly complex services, and deaths, which are sensitive to primary care. Another aggravating factor is the annual readjustment of drug prices, authorized by the Drug Market Regulation Chamber (CMED). In the period covered by the survey, there was an average 18.24% increase in the price of medicines, which represents a loss of purchasing power on the part of the municipalities for this purpose, not accompanied by compatible increases in the counterparts.

The increase in spending on medicines was proportionally higher among municipalities with a lower MHDI. This result is particularly noteworthy because, in 2019, Ordinance No. 3,193 began to differentiate the transfer amounts, contributing more to these municipalities. The results, however, show that this measure did not have a verifiable positive impact, as the amounts invested in 2020 represent an even higher percentage of own costs than in previous years. Those with the worst indicators (MHDI) suffered the most from the impact of the reduction in the federal contribution to the purchase of medicines, falling by 32% in municipalities with a population of 25,001 to 50,000 inhabitants between 2016 and 2020. In municipalities with a population of 50,001 to 100,000 inhabitants with a very high MHDI, however, there was an increase of 3% in the weight of the federal contribution.

According to a study by Faraco^28^, the municipalities with the lowest social and economic development (indicated by the MHDI) are those with the lowest PS management capacity (including technical and organizational activities, structure and inter-institutional relations). The municipalities with the highest MHDI values are those with the highest density of pharmacists per 10,000 inhabitants in the municipal health network workforce and in these municipalities with a higher proportion of pharmacists in the services, health unit users reported better access to and more information about their medicines^29^. Therefore, it can be inferred that the most vulnerable municipalities in socio-economic terms have the lowest *per capita* value for the purchase of medicines and also the most precarious conditions for managing these resources and offering pharmaceutical services to residents, making up an important picture of unequal access to health care among Brazilian citizens.

The majority of resources transferred to PS are dedicated to the purchase of medicines, and in amounts below the growing needs, as shown. Only one strategy for transferring funds from the federal government to municipalities for structuring PS has been implemented - the QualifarSUS Program. However, the transfers are not automatic but depend on the action of the municipalities in sending data to the MS on a quarterly basis. The municipalities with the lowest MHDI are the ones that have most often failed to send data and, therefore, failed to receive funding to structure municipal PS^30^, aggravating the inequality between municipalities.

This study also found that municipalities in the Northeast and North were more dependent on transfers from the MS and the states in the years analyzed. Municipalities in the North and Northeast invested less than the national average in medicines between 2010 and 2019, when the values are corrected by the IPCA, as shown by Silva et al.^5^. Pontes et al.^6^ had also shown that the North and Northeast regions had the highest number of municipalities with the lowest investment in medicines between July 2013 and June 2014. This is in line with investments in health, where there are major regional disparities resulting in disparities in access to health services and, consequently, in health outcomes. According to Massuda et al.^31^, the poorest regions and the most disadvantaged socio-economic population groups are the most affected by the ways in which primary care is financed. The differences in the structure and workforce of municipal PS between the regions were clearly explained by the National Survey on Access and Rational Use of Medicines, which leads to the inference that the lower investments in the purchase of medicines in primary care shown here are directly related to the reduced capacity of these municipalities to offer not only medicines, but also pharmaceutical services for the best use of these resources.

One of the strategies to increase the population’s access to medicines in primary health care, the Farmácia Popular Program, has collaborated extensively as another way of accessing these medicines, funded solely by the MS. For health conditions such as diabetes and hypertension, almost 50% of patients report having already obtained medicines from pharmacies affiliated with the program^32^, which justifies its great social recognition. However, by 2022, the program had mainly reached the municipalities with the largest populations, while among the smaller population groups, the proportion of municipalities with partner pharmacies grew strongly as the MHDI increased. Therefore, for this to be a more effective strategy to help overcome the inequality in access to medicines among Brazilian municipalities, it is necessary to develop ways to expand the program in the most vulnerable municipalities, which has been implemented recently.

However, this study has limitations. Data provided by the municipalities to SIOPS was analyzed, such as total spending on health and the percentage of spending on medicines, which, being self-declaratory in nature, could not be confirmed. Also due to this self-declaratory nature, it was not possible to differentiate which medicines are purchased by the municipalities, as well as the quantity of medicines for primary care purchased directly by the MS and the states and passed on to the municipalities. It is estimated that most municipalities purchase medicines for primary care or the CBAF. However, it is known that municipal managers can decide to purchase other medicines with their own resources, as described by Faleiros et al.^9^, or are obliged to purchase them to comply with court orders. It was also not possible to obtain data on investments actually made by the states for the CBAF and the study was based on an estimate of compliance with the minimum investment defined by Ordinance.

The findings of this study show that inequalities in the ability of municipalities to ensure access to medicines are widening, especially among the most vulnerable municipalities, increasing the risk of illnesses and deaths from primary care-sensitive diseases. There is an urgent need to implement strategies to halt the progress of this process of distancing between the conditions of supply of primary care medicines between municipalities, especially in the most vulnerable regions. The differentiation of the transfer by MHDI implemented in 2019 was not enough to curb the advance of inequities, suggesting the need for more complex actions, beyond restoring the transfer of financial resources, which are necessary and provided for in the organization of the SUS.

## CONCLUSION

It was concluded that there was an increase in the proportion of investments made by municipalities for the purchase of medicines, in relation to transfers from the Federal Government, between 2016 and 2020. This has led to an increase in inequalities in the ability of municipalities to ensure access to medicines, especially among the most vulnerable municipalities, further increasing the risk of illnesses and deaths from primary care-sensitive diseases.
