# Supplementary material for: The financing of medicines in Brazilian municipalities: whose responsibility is it?
Source: Rev Saude Publica. 2024 Nov 25;58:48. doi: 10.11606/s1518-8787.2024057005565 (PMC11548910; doi:10.11606/s1518-8787.2024057005565)
Supplement: Translation

# Translation: O financiamento dos medicamentos nos municípios do Brasil: de quem é a responsabilidade?

**Authors:** Wendell Rodrigues Oliveira da Silva, Rodrigo Fonseca Lima, Ivanessa Thaiane do Nascimento Cavalcanti, Rafael Santos Santana, Silvana Nair Leite

**Affiliations:** I Universidade de Brasília. Programa de Pós-graduação em Ciências Farmacêuticas. Brasília, DF, Brasil; II Universidade Federal de Santa Catarina. Programa de Pós-graduação em Assistência Farmacêutica. Florianópolis, SC, Brasil

**Keywords:** Assistência Farmacêutica, Financiamento de Capital, Atenção Primária à Saúde, Medicamentos Essenciais, Cidades

## Abstract

**OBJETIVO:**

Analisar os investimentos realizados em medicamentos pelos entes federados e as assimetrias desses investimentos de 2016 a 2020 que podem representar impactos na oferta e no acesso desses medicamentos no SUS.

**MÉTODOS:**

Trata-se de um estudo exploratório, retrospectivo, para identificar quem são os principais entes responsáveis pelo investimento em medicamentos da Atenção Básica dos municípios, a evolução, as contrapartidas e as diferenças regionais deste investimento entre 2016 e 2020.

**RESULTADOS:**

Os valores gastos com medicamentos pelos municípios brasileiros foram maiores que a contrapartida para o CBAF do MS ou do MS + Estado em todos os anos analisados. As médias percentuais de repasses de recursos federais e de gastos municipais variam conforme região do Brasil. A média do valor *per capita* investido em medicamentos pelos municípios aumentou entre 2016 e 2020 (aplicada a deflação), sendo maior o impacto para os municípios de menores IDHM. O programa Farmácia Popular atinge principalmente os municípios dos maiores portes populacionais e de maiores IDHM, não sendo suficiente, portanto, para enfrentar as iniquidades de acesso apontadas.

**CONCLUSÕES:**

Houve ampliação das desigualdades na capacidade dos municípios para assegurar o acesso aos medicamentos, principalmente entre os municípios mais vulneráveis, acumulando ainda mais riscos de agravos e mortes por doenças sensíveis à atenção primária.

## INTRODUÇÃO

Garantir acesso aos medicamentos considerados essenciais é fundamental para a resolutividade das ações de saúde na atenção básica. Para a Organização Mundial da Saúde (OMS), a falta de acesso a medicamentos causa uma cascata de miséria e sofrimento e até mortes por doenças que são preveníveis ou curáveis^1^.

O financiamento da aquisição de medicamentos é tema de amplo interesse em todos os países e diferentes sistemas de saúde^2^. O Sistema Único de Saúde (SUS) tem a responsabilidade pelo acesso a medicamentos, estruturado por Componentes da Assistência Farmacêutica (AF). O Componente Básico da Assistência Farmacêutica (CBAF) destina-se à aquisição de medicamentos e insumos previstos nos Anexo I e Anexo IV da Relação Nacional de Medicamentos (Rename), incluindo-se aqueles relacionados aos agravos e programas de saúde da atenção primária.

Os recursos para o custeio do CBAF são compartilhados de forma tripartite, sendo a gestão destes recursos de responsabilidade municipal. Os repasses de valores fixados *per capita*, do Ministério da Saúde (MS) e dos estados para o fundo municipal de saúde, têm seus valores definidos em Portarias do MS. Em 2017, o valor definido para repasse do MS foi de R$ 5,58 por habitante/ano^3^. Já em 2019 foi publicada a Portaria nº 3.992/2017, que modificou a forma de financiamento e a transferência dos recursos para as ações e os serviços de saúde em dois blocos: o Bloco de Custeio das Ações e Serviços de Saúde e o Bloco de Investimento na Rede de Saúde. O financiamento do CBAF continuou sendo definido por Portaria específica. Neste ano, passou a ser considerado o Índice de Desenvolvimento Humano Municipal (IDHM) para a definição dos valores a serem repassados pelo MS para os municípios para o financiamento do CBAF, que variam de R$ 5,85 a R$ 6,05 e ainda estão vigentes até 2023 sem reajuste^4^. O MS é responsável, portanto, por cerca de 50% do valor mínimo estipulado para o custeio dos medicamentos disponibilizados pelos municípios. Os estados e municípios têm a responsabilidade por parcelas menores, *per capita*, para composição do orçamento total do CBAF.

No Brasil é possível verificar assimetrias de investimentos *per capita* em AF: de 2010 a 2019, nas regiões Centro-Oeste, Sudeste e Sul, houve aumento nesse valor, já as regiões Nordeste e Norte investiram menos que a média nacional e apresentaram recuo no valor do investimento *per capita* em medicamentos^5^. A menor aplicação de recursos financeiros da AF em algumas regiões já foi identificada e motiva preocupação, o que não gerou, até momento, políticas específicas capazes de mitigar as iniquidades^6^. Entre 2005 e 2009, observou-se aumento global de 61,6% nos recursos do SUS para a compra de medicamentos; neste período, os estados e o Distrito Federal aumentaram em 112,4% o volume de recursos próprios alocados no financiamento da aquisição de medicamentos, enquanto os municípios registraram, no Orçamento Públicos em Saúde (SIOPS), aumento de 22,7%^7^. O valor investido em 2009 ainda foi considerado menor que o valor *per capita* investido em medicamentos em países como Canadá e Itália^8^.

Pontes et al.^6^, analisando os dados registrados pelos municípios na Base Nacional de Dados das Ações e Serviços de Assistência Farmacêutica, identificaram que os da Região Sudeste, em média, aplicaram um valor por habitante/ano maior e adquiriram mais itens do que os das demais regiões. No entanto, o envio de dados qualificáveis aconteceu em apenas 17% dos municípios brasileiros.

É também importante considerar que, em pesquisa de amostra nacional de 600 municípios^9^, 35,4% dos secretários municipais de saúde afirmaram utilizar recursos de medicamentos do CBAF para cobrir demandas de outros medicamentos, e apenas 9,7% declararam que esses recursos são suficientes para atender à demanda da atenção básica.

A estruturação da AF nos municípios brasileiros, além dos investimentos diretos na aquisição de medicamentos, apresenta também condições desiguais entre as regiões geográficas, incluindo estrutura física, de força de trabalho, de capacidade de gestão^9^, resultando em desigualdade na oferta de medicamentos disponibilizados para a população entre estas regiões^14^. Ainda assim, 59,8% dos usuários das unidades de atenção básica referiram, em 2015, ter acesso total aos medicamentos prescritos no SUS, demonstrando o grande impacto social da AF na atenção básica^15^.

O financiamento da aquisição de medicamentos e a garantia da acessibilidade na atenção básica são questões ainda mais críticas na atualidade, pois dados da Pesquisa Nacional de Saúde de 2019 revelaram que apenas 30,5% das pessoas obtiveram seus medicamentos no SUS, na média nacional, sendo a Região Sul a com maior acesso^16^. Este resultado é especialmente preocupante por revelar que o acesso das pessoas aos medicamentos pode estar diminuindo nos últimos anos, considerando que, em pesquisa nacional de 2014, 47% da população brasileira declarou ter obtido seus medicamentos prescritos para doenças crônicas nas unidades do SUS^17^. Nos últimos anos também foi autorizado reajuste de 18,24% nos preços dos medicamentos pela Câmara de Regulação do Mercado de Medicamentos (2016 a 2020) em paralelo à recessão econômica que atinge a população e leva à maior dependência em relação ao SUS.

Ao longo da história da consolidação do SUS, a AF se constitui como política e serviço fundamental e com grande impacto para a população brasileira^18^. Além da regulamentação do financiamento dos medicamentos por componentes, no SUS, foi instituído o Programa Farmácia Popular para ampliar a capacidade de promover acesso aos medicamentos para condições prevalentes, o qual se consolidou como importante estratégia de garantia de direitos para a população. Na atualidade, em que o modelo de atenção básica à saúde e seu financiamento passam por modificações contumazes e preocupantes^19^, e que se vislumbra a retomada das políticas públicas para equidade e universalidade do acesso à saúde no país, é preciso analisar a forma como, de fato, tem sido financiada a aquisição de medicamentos para as condições de saúde atendidas nos serviços de atenção básica, buscando identificar possíveis iniquidades, distorções e condições inadequadas de financiamento para a população, o acesso aos medicamentos e as ameaças à sustentabilidade dos municípios como gestores da atenção básica.

O repasse de recursos para a gestão municipal da saúde é um fator crítico a ser analisado. Questões como a forte desigualdade de renda e desenvolvimento social, as grandes extensões territoriais e as regiões de difícil acesso e contrastes regionais elevam o grau de dificuldade para prestação de serviços de atenção básica, incluindo a AF20. Este estudo teve por objetivo analisar os recursos empregados na aquisição de medicamentos da atenção básica à saúde entre as responsabilidades dos entes federados em um período recente de cinco anos (2016, 2018 e 2020), que compreende a época em que foi aplicado um novo cálculo para o repasse de recursos federais. O estudo contempla a totalidade dos municípios brasileiros a partir dos registros no SIOPS, analisando as características dos municípios como gestores destes recursos e o impacto das contrapartidas pactuadas na tripartite.

## MÉTODOS

Trata-se de um estudo exploratório retrospectivo, no qual se identifica e se compara a evolução dos valores das contrapartidas do CBAF pelo MS, estados e municípios, na série temporal de 2016, 2018 e 2020. O período contempla os repasses ocorridos antes e depois das correções do valor da contrapartida por parte do MS ocorridas em 2017^3^.

Os dados referentes aos valores da contrapartida do MS foram coletados do site do Fundo Nacional de Saúde (FNS)^21^ através do demonstrativo das transferências de recursos realizadas por esse órgão, por bloco de financiamento. Dos 5.568 municípios pesquisados, identificou-se que apenas o município de Cárcere/MT não recebeu os repasses dos recursos da contrapartida do MS nos anos pesquisados e Brasília não foi incluída na pesquisa, por não ser município. Todos os recursos descentralizados pelo FNS para aquisição de medicamentos do CBAF (diretamente aos municípios ou aos estados para aquisição centralizada) foram contabilizados para cada município de destino. Buscou-se identificar a regularidade do repasse da contrapartida do CBAF dos estados aos municípios de várias formas (em contato com setores do MS, Conselho Nacional de Secretários de Saúde – Conass e Conselho Nacional de Secretarias Municipais de Saúde – Conasems), porém, não foi encontrada uma fonte segura de informação sobre este repasse para todos os estados brasileiros. Dessa forma, considerou-se no estudo que todo recurso referente à contrapartida dos estados, previsto pela normativa vigente em cada ano, foi repassado.

Os dados referentes à população, gasto total em saúde e percentual de gasto com medicamentos foram extraídos do Sistema de Informações sobre SIOPS^22^, que constitui uma ferramenta para o monitoramento do cumprimento do dispositivo constitucional que determina, em orçamento, a aplicação mínima de recursos em ações e serviços públicos de saúde:

- População, que representa a população do município divulgada pelo Instituto Brasileiro de Geografia e Estatística (IBGE), base alimentada pelo censo e suas estimativas anuais;
- Despesa total em saúde (D.Total Saúde), que representa os gastos totais com saúde dos entes durante o período avaliado. Esse indicador resulta do gasto em saúde, por habitante, advindo de todas as fontes, quer sejam impostos, transferências do SUS (União, estados e outros municípios), operações de créditos e outras;
- Participação percentual da despesa com medicamentos na despesa total com saúde que representam a participação dos gastos com medicamentos em relação ao total dos gastos com Saúde (%D.com Medicamentos).

Foram elaboradas planilhas contemplando todos os municípios brasileiros, permitindo a análise a partir de frequências absolutas e relativas no intervalo temporal examinado com dados secundários oriundos do FNS e do SIOPS. Foi utilizado, também, o programa estatístico R*studio* para a limpeza dos *outliers* do banco de dados, conforme o diagrama de caixa (BoxPlot) e a percentagem (1% e 99%).

Para cálculo do valor investido em medicamentos pelos municípios, foram utilizados os dados referentes às despesas totais em saúde multiplicando-se pela participação percentual da despesa com medicamentos na despesa total com saúde.

Foram calculados os valores *per capita* do gasto em medicamentos por região, considerando a média dos anos de análise; a média da percentagem de repasse de recurso do MS e do recurso do MS mais a contrapartida estadual no gasto com medicamento; a média do valor dos três anos do repasse de recurso do MS e do recurso do MS mais a contrapartida estadual no gasto com medicamento e a média do valor gasto com medicamento em 2016, 2018 e 2020.

Fez-se a atualização monetária dos valores gastos com medicamentos e repasses federal e estadual aos municípios. Esses valores foram deflacionados para dezembro/2021, empregando-se a variação anual do Índice Nacional de Preços ao Consumidor Amplo (IPCA), calculado pelo IBGE e obtido do seu site ajustado pela estimativa de população para cada ano analisado. A opção pelo uso deste deflator baseou-se na Lei nº 10.742/2003, que estabeleceu as normas de regulação do setor farmacêutico e definiu este índice para fins de ajuste nos preços dos medicamentos no país.

Devido à natureza do SIOPS ser autodeclaratória, não foi possível a identificação, em relação às despesas dos municípios, de quais valores foram quitados com recursos oriundos da União, do estado ou do próprio município.

Os dados relativos a farmácias cadastradas atendendo o Programa Farmácia Popular foram extraídos do sistema Farmácia Popular – Gestão 2.0, sistema responsável por integrar as farmácias credenciadas ao MS.

Em relação ao porte populacional, utilizou-se do modelo de estrato desenvolvido pelo Programa Qualifar-SUS, programa do MS que apoia as atividades da AF nos municípios brasileiros, naqueles de até 25.000 habitantes, de 25.001 a 50.000, de 50.001 a 100.000, de 100.001 a 500.000 e municípios acima de 500.000 habitantes^23^.

O índice IDHM estima o desenvolvimento humano de um município e classifica os municípios em cinco faixas: muito baixo (de 0,000 a 0,499), baixo (0,500 a 0,599), médio (0,600 a 0,699), alto (0,700 a 0,799) e muito alto (de 0,800 em diante)^24^.

Foi empregado o teste de significância T de *Student* ou teste T, considerando significativos os resultados de p < 0,05 (5%). Nesta amostra foi utilizado o teste T para duas amostras independentes (ou não pareadas) para comparar as médias de duas amostras independentes.

## RESULTADOS

No que se refere ao financiamento para a aquisição de medicamentos, os valores gastos com medicamentos pelos municípios brasileiros foram maiores do que os valores de contrapartida do MS ou da contrapartida do MS + Estado (para o financiamento do CBAF) em todos os anos analisados. A mediana dos gastos com medicamentos pelos municípios (n = 3.740) passou de R$ 409.281,59 em 2016 a R$ 577.522,66 em 2020 (n = 4.152), configurando um aumento de 41,1% na mediana de gastos. Em relação à mediana de repasse financeiro do MS e do estado aos municípios, houve uma redução de 3,19% e 15,01%, respectivamente, quando comparados os anos de 2016 e 2020, e aplicado reajuste inflacionário (Figura 1).

**Figura 1:** Mediana dos gastos (R$) com medicamentos pelos municípios brasileiros e de repasse feito pela União (MS) e Estados nos anos de 2016, 2018 e 2020.

As médias percentuais de repasses de recursos federais com e sem contrapartida estadual e de gastos municipais considerando os três anos analisados variam conforme a região do Brasil (Figura 2). A média percentual nacional de gastos municipais foi de 69, variando de 59 na Região Nordeste até 76 na Região Centro-Oeste.

**Figura 2:** Média percentual (%) de repasses de recursos e de gastos municipais por região.

Na Tabela 1 pode-se observar que a porcentagem média de recurso repassado pelo MS e estados sobre a média do valor total investido em medicamentos pelos municípios brasileiros em 2016 e 2020, por estrato populacional e IDHM, diminuiu significativamente (p < 0,05) na grande maioria dos municípios. Somente não houve queda da representatividade do repasse do MS nos municípios com 50.001 a 100.000 habitantes e IDHM muito alto, como apresentado na Tabela 1.

**Tabela 1:** Análise da média da percentagem de recurso repassado pelo MS sobre o valor total investido em medicamentos pelos municípios brasileiros em 2016 e 2020, por estrato populacional e IDHM.

Estrato populacional (habitantes)	IDHM	n	2016		2020		Diferença de 2016 para 2020	
			n	% sobre total	n	% sobre total	%	p-value
Até 25.000	Muito baixo	25	16	42	16	29	13	0,024
	Baixo	1.111	599	48	655	27	21	0,0398
	Médio	1.778	1.166	33	1.336	22	11	0,016
	Alto	1.209	969	23	1.064	18	5	0,0041
	Muito alto	2	2	23	2	7	16	-
25.001 a 50.000	Muito baixo	9	6	51	7	19	32	0,0142
	Baixo	204	125	65	135	35	30	0,0248
	Médio	269	154	49	179	33	16	0,0285
	Alto	279	228	34	247	28	6	0,0035
	Muito alto	3	2	17	2	12	5	0,0198
50.001 a 100.000	Muito baixo	2	0^a^	-	1	74	-	-
	Baixo	47	30	60	33	39	21	0,0198
	Médio	124	65	60	78	38	22	0,0381
	Alto	176	136	37	149	30	7	0,0058
	Muito alto	5	5	22	4	25	-3	0,0051
100.001 a 500.000	Muito baixo^a^	0	0	-	0	-	-	-
	Baixo	5	3	58	2	37	21	0,0634
	Médio	61	38	54	44	34	20	0,0202
	Alto	191	144	37	150	35	2	0,0082
	Muito alto	20	16	25	18	20	5	0,0041
Acima de 500.000	Muito baixo^a^	0	0	-	0	-	-	-
	Baixo^a^	0	0	-	0	-	-	-
	Médio	1	0^a^	-	1	37	-	-
	Alto	34	24	50	19	43	7	0,0283
	Muito alto	13	12	44	10	34	10	0,0057

IDHM: Índice de Desenvolvimento Humano Municipal; n: quantidade de municípios.

^a^ Sem município para a classificação específica de IDHM e/ou para o ano.

Como pode ser observado na Tabela 2, a porcentagem de municípios que possuem pelo menos uma farmácia credenciada ao Programa Farmácia Popular aumenta com o porte e IDHM entre os estratos apresentados. Ademais, observa-se aumento estatisticamente significativo de média do valor *per capita* investido em medicamentos pelos municípios entre 2016 e 2020 (aplicada a deflação) em todos os estratos, com exceção nos municípios com mais de 500.000 habitantes com IDHM alto. Com exceção dos municípios de até 25.000 habitantes, aqueles com menores IDHM tiveram os maiores aumentos de valores investidos no mesmo período (Tabela 2).

**Tabela 2:** Porcentagem de municípios com farmácias credenciadas ao Programa Farmácia Popular e média do valor *per capita* investido em medicamentos em 2016 e 2020 pelos municípios brasileiros por estrato populacional e IDHM.

Dados municipais			Municípios com FP	Dados relacionados ao valor per capita					
Estrato populacional (habitantes)	IDHM	n	2020	2016		2020		Diferença de 2016 para 2020	
			%	n	Média (R$)	n	Média (R$)	%	p-valor
Até 25.000	Muito baixo	25	20	16	43,95	16	63,06	43	0,024
	Baixo	1.111	49	599	42,06	655	65,01	55	0,0398
	Médio	1.778	78	1.166	53	1336	75,68	43	0,016
	Alto	1.209	92	969	77	1.064	94,38	23	0,0041
	Muito alto	2	100	2	55,29	2	146,83	166	-
25.001 a 50.000	Muito baixo	9	22	6	28,93	7	60,13	108	0,0142
	Baixo	204	70	125	26,27	135	50,01	90	0,0248
	Médio	269	93	154	31,36	179	43,14	38	0,0285
	Alto	279	100	228	44,86	247	56,64	26	0,0035
	Muito alto	3	100	2	57,2	2	72,93	28	0,0198
50.001 a 100.000	Muito baixo	2	0	0	-	1	12,29	-	-
	Baixo	47	81	30	23,78	33	40,98	72	0,0198
	Médio	124	97	65	27,53	78	36,55	33	0,0381
	Alto	176	100	136	39,28	149	49,04	25	0,0058
	Muito alto	5	100	5	55,6	4	64,05	15	0,0051
100.001 a 500.000	Muito baixo^a^	0	0	0	-	0	-	-	-
	Baixo	5	100	3	16,88	2	26,57	57	0,0634
	Médio	61	100	38	33,21	44	52,39	58	0,0202
	Alto	191	100	144	35,68	150	40,17	13	0,0082
	Muito alto	20	100	16	44,27	18	52,23	18	0,0041
Acima de 500.000	Muito baixo^a^	0	0	0	-	0	-	-	-
	Baixo^a^	0	0	0	-	0	-	-	-
	Médio	1	100	0	-	1	24,59	-	-
	Alto	34	100	24	35,1	19	30,32	-14	0,0283
	Muito alto	13	100	12	36,05	10	44,12	22	0,0057

FP: farmácias credenciadas ao Programa Farmácia Popular; IDHM: Índice de Desenvolvimento Humano Municipal; n: quantidade de municípios.

^a^ Sem município para a classificação específica de IDHM e/ou para o ano.

## DISCUSSÃO

O valor da mediana do repasse da contrapartida do MS de 2016 a 2020 teve uma queda de 3,19%, mostrando o não acompanhamento dos investimentos em medicamento por parte desse Ministério nos gastos dos municípios. No mesmo período, a média do valor *per capita* investido em medicamentos pelos municípios aumentou significativamente (aplicada a deflação) em todos os estratos populacionais e, especialmente, entre aqueles com menores IDHM. Como resultado, o financiamento deste componente tem sido ampliado com recursos próprios dos municípios, que arcaram 67%, em média, de todos os recursos aplicados na compra de medicamentos no âmbito municipal entre 2016 e 2020. Considerando, no entanto, o aumento geral do investimento em medicamentos pelo MS no mesmo período, é importante também ponderar que este realiza a aplicação direta de recursos para aquisição de medicamentos e insumos para atenção básica, como as insulinas e os medicamentos relacionados ao Programa Saúde da Mulher. O Programa Farmácia Popular do Brasil, ainda que não compute nos valores empregados nos Componentes da Assistência Farmacêutica, na prática também colabora para aumentar o acesso dos medicamentos padronizados para atenção básica dos municípios contemplados. Ainda assim, o aumento expressivo dos investimentos diretos realizados pelos municípios indica aumento da responsabilidade destes entes federados para a garantia do direito de acesso aos medicamentos.

O comportamento do financiamento da atenção básica tem sido amplamente pressionado pelo aumento da carga dos municípios nesta conta. Araújo^25^ já demonstrou que, em um cenário que combina descentralização e subfinanciamento da política pública de saúde, os municípios brasileiros são os que mais contribuem com o setor saúde em termos proporcionais a sua arrecadação e aumentam significativamente seus gastos no setor, alocando proporção das receitas próprias em níveis superiores aos determinados constitucionalmente. Descrição recente aponta que os valores de investimento geral em saúde pela União aumentaram 30,7% em 2019 quando comparados com 2012; os estados apresentaram uma pequena queda de investimentos durante o mesmo período, enquanto os municípios apresentaram maior incremento (32,8% de 2010 a 2019)^5^. No estudo de De Seta et al.^26^, foi identificado que, de 1991 a 2017, a participação da União no financiamento do SUS reduziu de 73% para 43%, e foi acompanhada do aumento da alocação de recursos, principalmente municipal, levando a uma maior carga orçamentária aos municípios.

O atual estudo demonstra que o comportamento do financiamento da compra de medicamentos da atenção básica neste período entre os entes federados apresentou maior discrepância do que a observada no financiamento geral da saúde. A contribuição dos municípios apresentou um aumento percentual em relação à contrapartida da União e estados ainda maior do que vem sendo observado no financiamento da saúde, apesar dos reajustes de valores de repasse para o CBAF normatizados em 2017 e 2019.

Bruns et al.^27^ apresentam dados, no entanto, que podem significar agravante aos resultados aqui apontados, ao indicar a falha nos repasses de contrapartida estadual em alguns estados, evidenciando a baixa contribuição dessa esfera para com os municípios. Portanto, considerando que para o presente estudo foi adotado o pressuposto de que os estados efetivaram suas contrapartidas normatizadas (em valores ou em medicamentos), os dados apresentados podem ser ainda mais alarmantes para os municípios. Como um ciclo vicioso, a falta desses recursos pode influenciar o aumento da demanda de serviços de média e alta complexidade, e mortes, sensíveis à atenção primária. Outro agravante é o reajuste anual de preços dos medicamentos, autorizados pela Câmara de Regulação do Mercado de Medicamentos (CMED). No intervalo da pesquisa, houve um total de 18,24% de reajuste médio nos preços dos medicamentos, o que configura a perda do poder aquisitivo dos municípios para este fim, não acompanhado por reajustes compatíveis nas contrapartidas.

O aumento do gasto próprio para o financiamento dos medicamentos foi proporcionalmente maior entre os municípios de menor IDHM. Este resultado chama especial atenção, pois, em 2019, a Portaria n^o^ 3.193 passou a diferenciar os valores de repasse, contribuindo mais para estes municípios. Os resultados, no entanto, revelam que esta medida não logrou impactos positivos verificáveis, pois os valores investidos em 2020 representam uma porcentagem de custeio próprio ainda mais elevada que nos anos anteriores. Aqueles com piores indicadores (IDHM) são os que mais sofreram o impacto da retração da contrapartida federal na compra de medicamentos, chegando a apresentar uma queda de 32% nos municípios com população de 25.001 a 50.000 habitantes entre 2016 e 2020. Já nos municípios com população de 50.001 a 100.000 habitantes com IDHM muito alto, no entanto, é identificado, inclusive, incremento de 3% no peso da contrapartida federal.

Os municípios com menor desenvolvimento social e econômico (indicado pelo IDHM), segundo estudo de Faraco^28^, são os que apresentam menor capacidade de gestão da AF (incluindo atividades técnicas e organizacionais, estrutura e relações interinstitucionais). Os municípios com maiores valores de IDHM são os que têm maior densidade de farmacêuticos por 10.000 habitantes na força de trabalho da rede municipal de saúde e nestes municípios com maior proporção de farmacêuticos nos serviços, os usuários das unidades de saúde referiram melhor acesso e mais informações sobre seus medicamentos^29^. Portanto, pode-se inferir que os municípios mais vulneráveis em termos socioeconômicos apresentam o menor valor *per capita* para aquisição de medicamentos e também as condições mais precárias para gerenciar estes recursos e ofertar serviços farmacêuticos para os munícipes, compondo um quadro importante de desigualdade de acesso à atenção à saúde entre os cidadãos brasileiros.

O repasse de recursos para AF é majoritariamente dedicado à aquisição de medicamentos, e em valores abaixo das necessidades crescentes, como demonstrado. Apenas uma estratégia de repasse de recursos do governo federal para os municípios para estruturação da AF foi implementada – o Programa QualifarSUS. No entanto, os repasses não são automáticos, mas dependentes de ação dos municípios no envio de dados para o MS trimestralmente. Entre os municípios de menores IDHM estão os que mais têm deixado de enviar dados e, portanto, deixado receber recursos de custeio para a estruturação da AF municipal^30^, agravando o quadro de desigualdade entre os municípios.

O presente estudo também identificou uma maior dependência dos municípios do Nordeste e do Norte em relação ao repasse do MS e dos estados nos anos analisados. Os municípios do Norte e Nordeste investiram menos que a média nacional em medicamentos entre 2010 e 2019, quando os valores são corrigidos pelo IPCA, conforme demonstrado por Silva et al.^5^. Pontes et al.^6^ já haviam também demonstrado que as regiões Norte e Nordeste foram as que apresentam o maior número de municípios com menor aplicação de recursos em medicamentos no período de julho de 2013 a junho de 2014. Tal fato se alinha aos investimentos em saúde, em que há grandes disparidades regionais resultando em disparidades no acesso aos serviços de saúde e, consequentemente, nos resultados de saúde. Para Massuda et al.^31^, as regiões mais pobres e os grupos populacionais socioeconômicos mais desfavorecidos são os mais prejudicados pelas formas de financiamento da atenção básica. As diferenças da estruturação e força de trabalho da AF municipal entre as regiões foram explicitadas claramente pela Pesquisa Nacional sobre Acesso e Uso Racional de Medicamentos, o que leva a inferir que os menores investimentos na compra de medicamentos na atenção básica aqui demonstrados estão diretamente relacionados com a capacidade reduzida destes municípios em ofertar não apenas medicamentos, mas também serviços farmacêuticos para o melhor uso destes recursos.

Uma das estratégias para ampliar o acesso da população aos medicamentos da atenção primária à saúde, o Programa Farmácia Popular, tem colaborado amplamente como mais uma via de acesso a estes medicamentos, com financiamento apenas do MS. Para condições de saúde como diabetes e hipertensão, perto de 50% dos pacientes relatam já ter obtido medicamentos nas farmácias conveniadas ao programa^32^, o que justifica o seu grande reconhecimento social. No entanto, o programa atingiu, até 2022, principalmente os municípios de maior porte populacional, dentre os grupos porte populacional menor, a proporção de municípios que contam com farmácias conveniadas cresce fortemente com o crescimento do IDHM. Portanto, para que esta seja uma estratégia mais efetiva para colaborar com a superação da iniquidade no acesso aos medicamentos entre os municípios brasileiros, é necessário desenvolver formas de ampliar o programa nos municípios mais vulneráveis, o que vem sendo implementado recentemente.

Este estudo, porém, tem limitações. Foram analisados dados fornecidos pelos municípios ao SIOPS, como os referentes ao gasto total em saúde e ao percentual de gasto com medicamentos, que, por serem de natureza autodeclaratória, não foram passíveis de confirmação. Também devido a essa natureza autodeclaratória não foi possível diferenciar quais medicamentos são adquiridos pelos municípios, assim como o quantitativo de medicamentos para atenção básica foi adquirido diretamente pelo MS e pelos estados, e repassado aos municípios. Estima-se que a maior parte dos municípios adquire os medicamentos relativos à atenção básica ou ao CBAF, no entanto, sabe-se que os gestores municipais podem decidir por adquirir outros medicamentos com recursos próprios, como já descrito por Faleiros et al.^9^, ou são obrigados a adquiri-los para atender mandados judiciais. Também não foi possível obter os dados de investimentos efetivamente aplicados pelos estados para o CBAF e o estudo se baseou na estimava do cumprimento do investimento mínimo definido por Portaria.

Os achados deste estudo mostram a ampliação das desigualdades na capacidade dos municípios para assegurar o acesso aos medicamentos, principalmente entre os municípios mais vulneráveis, acumulando ainda mais riscos de agravos e mortes por doenças sensíveis à atenção primária. É evidente a urgência na implementação de estratégias para frear o avanço deste processo de distanciamento entre as condições de oferta dos medicamentos de atenção básica entre os municípios, especialmente nas regiões mais vulneráveis. A diferenciação de repasse por IDHM implementada em 2019 não foi suficiente para dirimir o avanço das iniquidades, sugerindo a necessidade de ações mais complexas, para além da recomposição do repasse de recursos financeiros, necessários e previstos na organização do SUS.

## CONCLUSÃO

Conclui-se que houve ampliação na proporção dos investimentos dos municípios para a compra de medicamentos, em relação aos repasses da União, no período entre 2016 e 2020. Este fato gera aumento das desigualdades na capacidade dos municípios para assegurar o acesso aos medicamentos, principalmente entre os municípios mais vulneráveis, acumulando ainda mais riscos de agravos e mortes por doenças sensíveis à atenção primária.
